# From response-to-routine: embedding humanitarian supply evaluation in Singapore's health and community care systems

**DOI:** 10.3389/frhs.2026.1761596

**Published:** 2026-02-25

**Authors:** Huay Ling Tay, Chuan De Foo

**Affiliations:** 1 School of Business, Singapore University of Social Sciences, Singapore, Singapore; 2 Duke NUS Medical School, NUS Saw Swee Hock School of Public Health and National University Health System, Singapore, Singapore

**Keywords:** ageing population, climate adaptation, community care, health security, health systems resilience, humanitarian supply chain, Singapore

## Abstract

Singapore's ageing population and exposure to climate-related and pandemic risks demand a health system that is not only efficient but also inherently resilient to shocks and equitable in health service delivery. Policies need to address the current supply chain evaluations in public health to focus on cost and speed of healthcare delivery to close critical gaps in preparedness during times of sudden disruptions. Embedding a humanitarian perspective in public healthcare systems provides frameworks for resilience, equity, and sustainability. Yet these principles remain siloed from routine health governance. This policy brief argues for embedding humanitarian supply evaluation into Singapore's health and community care systems through a “Response-to-Routine” paradigm. We propose an implementable four-dimensional framework: Resilience & Equity, Climate & Sustainability, Governance & Collaboration, and Innovation & Learning, supported by scenario-based stress testing and community-level integration. This approach designed for policy level decision making ensures continuity of care for vulnerable populations, particularly older adults, while aligning with climate and health equity goals. Importantly, policymakers should institutionalise resilience audits and pilot integrated evaluation models in the existing healthcare landscape through a logistics and health systems lens.

## Introduction

Health systems face converging pressures: demographic ageing, climate volatility, and pandemic threats. These stressors expose fragilities in health systems that rely on lean, globally dependent supply chains. Fragmentation in these chains can manifest as delayed care, excess mortality, and widening inequities, making supply performance a critical determinant of health outcomes rather than a technical concern (1, 2, 4, 9). In recent years, humanitarian supply chains have evolved sophisticated evaluation frameworks to manage volatility, prioritising resilience, equity, and sustainability (1, 3).

Health systems policies need to be angled to address supply chain domains. Humanitarian supply chains in the context of public health refers to the strategic planning, management, and movement of essential supplies, resources, and services to populations affected by crises, disruptions, or vulnerabilities (4). Unlike traditional supply chain models that focus on cost and efficiency, humanitarian logistics prioritise resilience, equity, and sustainability from the outset of planning till delivery and evaluation. It encompasses processes for sourcing, storing, transporting, and distributing medical supplies, pharmaceuticals, personal protective equipment (PPE), and other critical resources, ensuring rapid response and continuity of care, especially for the most at-risk groups during emergencies such as pandemics, natural disasters, and climate-related events. These frameworks are designed to withstand volatility, adapt to changing circumstances, and maintain service delivery regardless of routine or surge conditions, aligning with principles of humanity, impartiality, and sustainability through mechanisms established during peacetime (1, 5, 9, 16).

However, policies looking into these principles remain largely absent from routine public health governance. Singapore, a high-density city-state with advanced health infrastructure and a rapidly ageing population, exemplifies this gap. Singapore's experience during SARS in 2003 and COVID-19 in 2020 highlighted vulnerabilities in global supply networks and the importance of rapid local adaptation to global level shocks (5). Healthier SG is a national initiative by the Singapore's Ministry of Health (MOH) focusing on preventive health. Singaporeans can take proactive steps to manage their health, prevent the onset of chronic diseases and have strong support to lead healthier lifestyles (2). It is a landmark initiative transforming the Singapore Healthcare System to one that place primary care at the forefront of the national healthcare system. While Singapore's national preventive health transformation initiatives, such as Healthier SG, strengthen preventive care and longitudinal chronic disease management for population health, they do not systematically embed resilience metrics into supply chain evaluation. This perspective paper argues for a paradigm shift: from *ad-hoc*, event-driven (sometimes even calendar-driven) audits to routine, system-integrated evaluation (1, 2, 17, 18). Embedding humanitarian principles into everyday health supply governance will transform supply chains from transactional functions into strategic pillars of health security (1, 2, 4, 6, 19).

### Robust supply evaluation is a critical public health imperative that demands strategic policy insight and governance

The World Health Organization recognises medicines and equipment supply as a core health system building block and emphasises the need for robust policy frameworks to ensure its long-term viability (21). Supply chains underpin essential health services, from pharmaceuticals and diagnostics to community-based care (17). Disruptions cascade into clinical and social consequences, particularly for older adults reliant on home care and chronic medication. In humanitarian contexts, supply evaluation is central to protecting life and equity during crises (18, 19). Public health systems, however, often treat supply performance as a back-office metric, disconnected from resilience and equity goals (2, 6, 13). In Singapore, this separation is evident in supply chain evaluations for initiatives like the recent Healthier SG national policy, which primarily focus on cost and efficiency but are increasingly being leveraged for broader population health outcomes and resilience. For example, Healthier SG tracks several indicators for general practitioner (GP) reimbursement purposes, such as adherence to medication regimens, completion of recommended screenings, and vaccination coverage. These indicators not only inform payment mechanisms but also serve as proxies for overall population health, as they reflect continuity of care and preventive health engagement (7). However, these metrics need to focus on other aspects, such as resilience and provider- and patient-centeredness, which remain lacking.

By including measures such as medication adherence, regular screening, and vaccine administration in the evaluation framework, Singapore's health system can monitor the effectiveness and reach of primary care services. These metrics indirectly contribute to resilience by ensuring that supply chains support timely access to essential medications and vaccines, facilitate follow-up care, and enable rapid response during disruptions. For instance, robust tracking of medication and vaccination rates allows the system to identify gaps in supply or delivery, which can then be addressed through improved procurement practices, surge inventory planning, and targeted support for vulnerable groups.

Critically, mandating health policies that integrate these indicators into routine evaluation helps align supply chain performance with equity goals by ensuring that all segments of the population, including older adults and those with chronic conditions, can receive appropriate preventive care and follow-up. This approach transforms supply chain metrics from mere operational concerns into strategic tools for advancing public health resilience, equity, and sustainability, as envisioned in Singapore's evolving health governance framework.

WHO's health system resilience frameworks emphasise robustness and agility in broad strokes but under-specify supply governance policies. Conversely, humanitarian logistics literature offers actionable indicators: redundancy, adaptability, and last-mile reliability that can be institutionalized within health systems (3, 6, 9). Integrating these principles reframe supply evaluation as a public health function, ensuring continuity of care during shocks and reducing vulnerability for high-risk groups (6, 11). Notably, Singapore's secure and resilient supply chains contributed to fewer disruptions in cold chain management, PPEs, and COVID-19 vaccines and medicines during the height of the pandemic, when nations were struggling to cope with the shortage of these essential medical supplies. For instance, the country's success in maintaining vaccine cold chains and in reliably providing COVID-19 medications demonstrated operational pragmatism and ensured timely access for vulnerable populations, even amid global shortages and supply chain volatility (1, 7, 11). This achievement underscores the critical role of institutionalising humanitarian logistics indicators within health systems to safeguard the continuity of essential care during periods of crisis.

Singapore's health system benefits from strong central stewardship and integrated networks that govern evidence back policy making practices. Healthier SG and community care initiatives provide a platform for resilience-building. However, COVID-19 revealed systemic fragility: early shortages of PPE and diagnostics underscored dependence on global supply chains (1, 5). While national strategies now emphasise resilience, formal evaluation mechanisms remain limited. During the COVID-19 pandemic, Singapore's healthcare procurement processes prioritised rapid acquisition of personal protective equipment (PPE) based on price and delivery speed (5, 8, 9). However, this approach initially overlooked the need for surge inventory to respond to sudden spikes in demand. It failed to ensure equitable distribution to all community care providers, including smaller nursing homes and home-care agencies (9, 13). As a result, some frontline workers in community settings experienced delays in receiving adequate PPE, leading to unnecessary nosocomial transmissions and, in turn, healthcare disruptions, highlighting vulnerabilities in both surge capacity and equity (5, 8, 9). Additionally, the rush to secure supplies from overseas vendors did not fully consider the environmental impact or sustainability of sourcing practices, including carbon footprints and long-term supplier partnerships (3, 11). This example underscores the need for procurement models that balance cost and efficiency with resilience, equity, and sustainability in Singapore's healthcare supply chains before disaster strikes (1, 2, 8).

Singapore's experience maintaining COVID-19, pneumococcal, and influenza vaccine cold chains during COVID-19, and managing supply disruptions during haze episodes and SARS underscores the need for planned resilience. This anticipation was seen through the Public Health Preparedness Clinics set up post-SARS epidemic, where PPEs and medications were made available to train general practitioners to conduct infection control measures when an outbreak was to occur. Singapore also has an unofficial emergency stock of essential medications, vaccines, and PPE to be unlocked when the time calls for it (9). For an ageing population that is predisposed to morbidities, these gaps translate into heightened risk during disruptions and necessitate forward logistical healthcare planning.

## Conceptual interface

Humanitarian principles, including Humanity, Impartiality, Sustainability align with health system goals of equity and continuity. Community resilience frameworks emphasise social capital and service continuity for vulnerable groups (6, 11, 12, 20). Integrating humanitarian evaluation into health governance means assessing whether supply chains can pivot from routine to surge without deprioritising older adults or sustainability (2, 9). This interface transforms vulnerability into a design parameter rather than an afterthought (2, 6). In fact, most research highlights the need to place the most vulnerable first when crises hit (10).

## Operational and policy-ready “response-to-routine” evaluation framework

We propose a four-dimensional framework for embedding humanitarian principles into Singapore's health and community care systems at the policy level: (1) Resilience & Equity, (2) Climate & Sustainability, (3) Governance & Collaboration, and (4) Innovation & Learning. Indicators include redundancy, adaptability, carbon footprint tracking, multi-stakeholder evaluation, and digital twin simulations (1, 2). Fundamentally, this aligns with planetary health principles, recognising that health system resilience must incorporate environmental sustainability to mitigate climate risks. For instance, public health services can adopt green procurement policies by sourcing medical supplies from vendors committed to reducing carbon emissions and using recyclable packaging materials. Hospitals and clinics can implement energy-efficient practices, such as switching to renewable energy sources and optimising water usage, thereby lowering their environmental footprint while maintaining care continuity. During supply chain disruptions, such as those seen during pandemics or haze episodes, integrating sustainability into logistics planning helps ensure that emergency responses do not exacerbate environmental degradation. Furthermore, community care providers can prioritise sustainable delivery methods, like electric vehicles for home healthcare visits, and promote telemedicine as a low-carbon alternative for routine consultations.

For clarity and practical application, this section presents the four-dimensional framework in a sequential and uniform structure for each dimension: Introduction, Reasons for Change, Implementation Pathways, and Expected Outcomes.

### Four-dimensional policy framework

#### Policy consideration 1. Resilience & equity

Introduction: Resilience safeguards the continuity of essential services during shocks, while equity ensures that older adults (given the global ageing population) and other vulnerable groups receive timely and appropriate care across routine and surge conditions (1, 2, 6, 9, 11).

Reasons for Change: COVID-19 and prior outbreaks exposed early shortages and uneven distribution of PPE and diagnostics, revealing dependencies on global suppliers (or a particular few nations) and the absence of embedded equity metrics in routine supply governance (5, 8, 9, 13).

Implementation Pathways: (a) institutionalise resilience audits in provider licensing; (b) diversify sourcing and establish surge inventory with rotation protocols; (c) formalise last-mile delivery contingencies for home- and community-care; (d) embed equity rules for allocation that prioritise high-risk groups during disruptions (1, 2, 6, 9, 11, 13).

Expected Outcomes: Reduced service interruptions to protect the continuity of medications and assistive supplies, and narrower gaps in outcomes for older adults and other vulnerable populations (6, 9–11).

#### Policy consideration 2. Climate & sustainability

Introduction: Climate-aware, low-carbon supply strategies strengthen health system resilience and align with planetary health and one health principles, reducing environmental externalities while maintaining care continuity (2, 6, 12).

Reasons for Change: Health supply chains contribute to emissions and waste; crises can magnify this footprint if sustainability is not embedded from the outset in procurement and logistics (6, 11, 12). Importantly, climate disruptions precipitate humanitarian disasters and crises.

Implementation Pathways: (a) adopt green procurement (supplier emissions disclosure, recyclable packaging); (b) expand regional/local sourcing where feasible; (c) track carbon and waste metrics alongside cost and service KPIs; (d) incentivise low-emission distribution (e.g., route optimisation, EV fleets for medical transport) and telehealth for suitable services (2, 6, 11–13).

Expected Outcomes: Lowered supply chain carbon footprint, reduced waste, strengthened continuity during haze or pandemic disruptions, and co-benefits for population and environmental health (6, 11, 12).

#### Policy consideration 3. Governance & collaboration

Introduction: Transparent, multi-stakeholder governance integrates hospitals, primary care, community providers, and social sectors into a coherent supply evaluation ecosystem (2, 6, 9, 10).

Reasons for Change: Fragmented oversight obscures bottlenecks at the community level and weakens equitable allocation during surge; shared situational awareness and clear roles are prerequisites for timely, fair decisions (6, 9–11).

Implementation Pathways: (a) co-design supply audits with community providers and caregivers; (b) publish periodic dashboards on availability, lead times, and equity metrics; (c) integrate resilience/equity criteria into funding and contracting; (d) conduct joint exercises to validate allocation rules and escalation protocols (2, 6, 9–11).

Expected Outcomes: Faster collective response, more transparent accountability, improved last-mile reliability, and strengthened trust across providers and the public to improve overall uptake (6, 9–11).

#### Policy consideration 4. Innovation & learning

Introduction: Continuous learning, analytics, and simulation (e.g., digital twins, scenario stress tests) enable proactive risk management and adaptive policies (10, 12, 14, 15).

Reasons for Change: Dynamic hazards and demand volatility require predictive insights beyond retrospective KPIs; systems need mechanisms to capture lessons and update protocols routinely (9, 14, 15).

Implementation Pathways: (a) deploy scenario-based stress testing across hospital–primary–community networks; (b) use near real-time dashboards for stock, lead times, and allocation; (c) test policy levers (e.g., alternative sourcing, allocation heuristics) in simulations before rollout; (d) establish feedback loops to revise procurement, licensing, and training (10, 12, 14, 15).

Expected Outcomes: Earlier detection of risks, more agile policy updates, and sustained performance under both routine and surge conditions (10, 14, 15).

**Figure 1:**
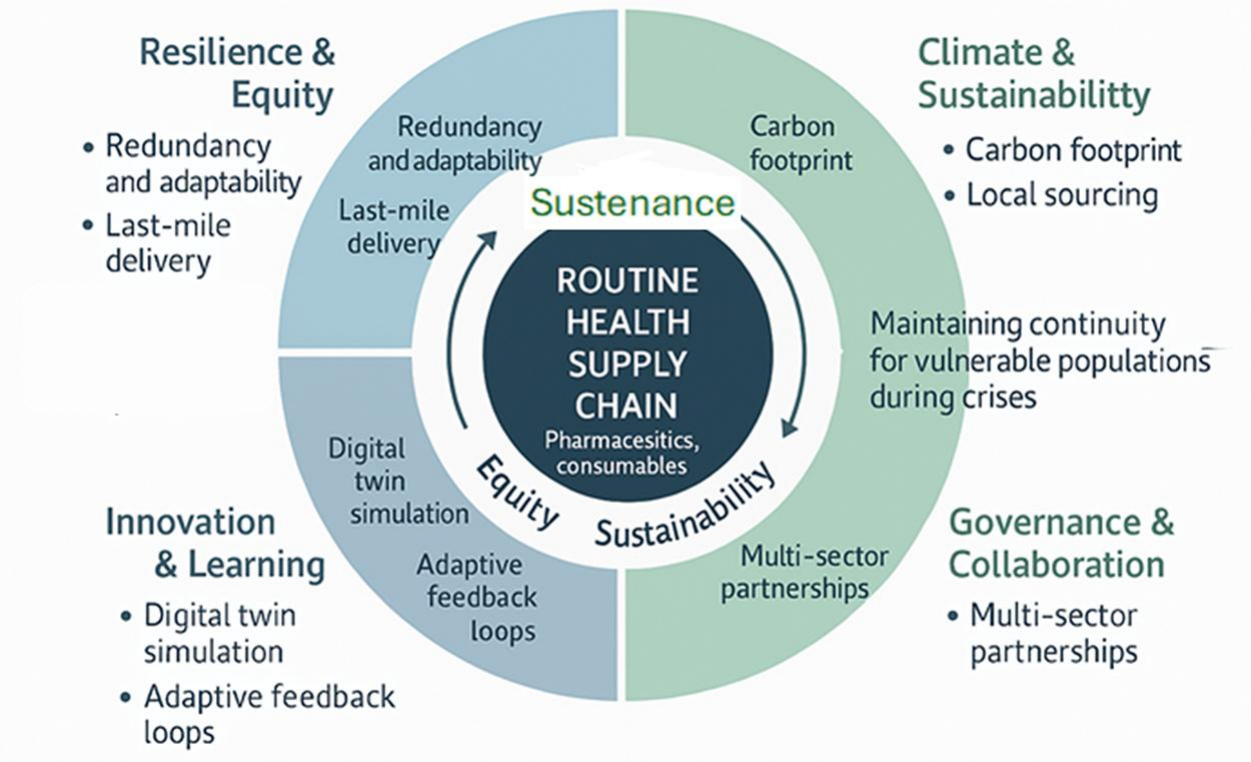
Response-to-routine evaluation framework for policy considerations.

By embedding these practices into public health service delivery, systems not only build resilience against shocks but also contribute to planetary health by safeguarding both human and environmental well-being. This framework ensures continuity of care for vulnerable populations and aligns with climate goals. See Table 1 and Figure 1 for details.

**Table 1:** Linking humanitarian supply concepts to public health and community resilience.

Dimension	Humanitarian concept	Health and community application	Primary beneficiaries
Resilience & equity (1, 10, 11)	Redundancy, adaptability, last-mile reliability (2, 11, 12)	Diversified sourcing, surge capacity, continuity of home-care supplies	Older adults, vulnerable groups
Climate & sustainability (2, 6)	Low-carbon logistics, local procurement, waste minimisation (11, 12)	Regional sourcing, green packaging, carbon footprint tracking	Environment, local Small and Medium Enterprises
Governance & collaboration (6, 10)	Multi-stakeholder evaluation, transparency (2, 6)	Inclusion of NGOs, social enterprises, and community hubs in supply audits	Community organisations, caregivers
Innovation & learning (10, 12, 13)	Digital twins, scenario-based stress tests (10, 14, 15)	Real-time supply chain simulation, feedback loops for policy updates	Health system planners, policymakers

## Policies need to place the most at risk first

Older adults depend on a web of supplies, including medications, nutrition, and assistive devices, delivered through hospitals, polyclinics, and home-care agencies. Disruptions to any node can precipitate avoidable hospitalisations and functional decline (1, 11, 13). For example, Dengue outbreaks in Singapore further stress supply chains for diagnostics and home-care kits, highlighting the need for robust evaluation to ensure continuity of care. Policies that embed humanitarian evaluation ensures continuity by mapping critical pathways, stress-testing performance under crisis scenarios (e.g., pandemic wave, haze event), and prioritising equity in allocation rules (7, 11). As seen in the most recent pandemic, the most vulnerable were the most at risk, often receiving the medicine and vaccine supplies last. These lived experiences places to the fore, the heightened need for all policies of the future to encapsulate health equity principles.

## Call to action & policy implications

The Agency for Integrated Care (AIC) is Singapore's national coordinating body for community and aged care services. AIC plays a pivotal role in coordinating aged-care services and can champion resilience audits and integrated evaluation models across community networks. Its mandate includes integrating care across hospitals, primary care, and community providers; allocating resources during crises; and building capacity for resilience. During COVID-19, AIC coordinated with various health entities, PPE distribution, telehealth deployment, and community care scaling, mitigating disruptions in essential services. AIC also manages digital platforms, such as the Community Care Management System, enabling real-time monitoring of service delivery and supply chain performance. These capabilities position AIC as a critical enabler for embedding resilience audits and humanitarian supply evaluation metrics into licensing and funding frameworks for nursing homes and home-care agencies.

We recommend policy makers explore the following when designing future supply chain policies: (1) Pilot Programme: Embed humanitarian logistics experts within MOH and AIC planning units to co-develop evaluation tools. (2) Mandatory Resilience Audits: Include resilience and equity metrics in licensing criteria for aged-care providers. (3) Research Agenda: Assess the cost-benefit of resilient vs. lean supply chains and develop composite resilience indices.

## Discussions and implications

We searched PubMed and Google Scholar for articles published between 2005 and 2024 using terms such as “humanitarian supply chain evaluation,” health system resilience,’ and “community care logistics.” Existing literature highlights robust frameworks for resilience and sustainability in humanitarian logistics, but these principles are rarely institutionalised within routine public health systems, particularly in ageing societies (2, 6).

### Added value of this study

This paper introduces a “Response-to-Routine” evaluation paradigm that embeds humanitarian supply evaluation principles, resilience, equity, and sustainability into Singapore's health and community care governance. It proposes a four-dimensional framework and actionable policy steps to bridge the gap between emergency response and everyday health supply management (1, 2).

### Implications of all available evidence

Integrating humanitarian evaluation into routine health supply governance can transform supply chains into strategic pillars of health security. For ageing, climate-vulnerable regions like the Western Pacific, this approach ensures continuity of care for older adults, strengthens preparedness for shocks, and aligns health systems with sustainability and equity goals.

## Policy and research implications

For Singapore and peers in the Health Services, embedding humanitarian supply evaluation into public health and community systems offers an opportunity to move from narrow emergency response to sustained resilience, equity and climate alignment (1, 2). Policymakers should treat humanitarian logistics not as an external adjunct but as a source of evaluative methods, metrics, and governance innovations that can be internalised into health and social policy, particularly in ageing societies (2, 6).

Further research is needed to co-develop context-appropriate indicators in collaboration with older adults, community partners, and humanitarian actors. Additionally, it is important to test models of integrated evaluation across hospital, primary care, and community care networks, and to assess how such integration influences health outcomes, social resilience, and environmental performance over time.

## Data Availability

The original contributions presented in the study are included in the article/Supplementary Material, further inquiries can be directed to the corresponding author.
